# International evaluation of the Microscale Audit of Pedestrian Streetscapes (MAPS) Global instrument: comparative assessment between local and remote online observers

**DOI:** 10.1186/s12966-021-01146-3

**Published:** 2021-06-30

**Authors:** Eric H. Fox, James E. Chapman, Abraham M. Moland, Nicole E. Alfonsin, Lawrence D. Frank, James F. Sallis, Terry L. Conway, Kelli L. Cain, Carrie Geremia, Ester Cerin, Griet Vanwolleghem, Delfien Van Dyck, Ana Queralt, Javier Molina-García, Adriano Akira Ferreira Hino, Adalberto Aparecido dos Santos Lopes, Jo Salmon, Anna Timperio, Suzanne E. Kershaw

**Affiliations:** 1 Urban Design 4 Health, Inc., Rochester, NY USA; 2 Department of Urban Studies and Planning, University of California San Diego, San Diego, CA USA; 3 Department of Family Medicine and Public Health (now Herbert Wertheim School of Public Health and Human Longevity Science), University of California San Diego, San Diego, CA USA; 4 Mary MacKillop Institute for Health Research, Australian Catholic University, Melbourne, Australia; 5 School of Public Health, The University of Hong Kong, Hong Kong, China; 6 Faculty of Medicine and Health Sciences, Department of Movement and Sport Sciences, Ghent University, Ghent, Belgium; 7 Department of Nursing, University of Valencia, Valencia, Spain; 8 Department of Teaching of Musical, Visual, and Corporal Expression, University of Valencia, Valencia, Spain; 9 Postgraduate Program in Health Sciences, Pontifical Catholic University of Parana, Curitiba, Brazil; 10 Postgraduate Program in Public Health, Federal University of Santa Catarina, Florianopolis, Brazil; 11 Institute for Physical Activity and Nutrition, School of Exercise & Nutrition Science, Deakin University, Geelong, Australia

**Keywords:** Microscale, Built environment, Pedestrian audit, Physical activity, Reliability, Remote data collection

## Abstract

**Objectives:**

The use of online imagery by non-local observers to conduct remote, centralized collection of streetscape audit data in international studies has the potential to enhance efficiency of collection and comparability of such data for research on built environments and health. The objectives of the study were to measure (1) the consistency in responses between local in-field observers and non-local remote online observers and (2) the reliability between in-country online observers and non-local remote online observers using the Microscale Audit of Pedestrian Streetscapes Global tool to characterize pedestrian-related features along streets in five countries.

**Methods:**

Consistency and inter-rater reliability were analyzed between local and non-local observers on a pooled database of 200 routes in five study regions (Melbourne, Australia; Ghent, Belgium; Curitiba, Brazil; Hong Kong, China; and Valencia, Spain) for microscale environmental feature subscales and item-level variables using the intraclass correlation coefficient (ICC).

**Results:**

A local in-field versus remote online comparison had an ICC of 0.75 (95 % CI: 0.68–0.80) for the grand total score. An ICC of 0.91 (95 % CI: 0.88–0.93) was found for the local online versus remote online comparison. Positive subscales yielded stronger results in comparison to negative subscales, except for the similarly poor-performing positive aesthetics/social characteristics.

**Conclusions:**

This study demonstrated remote audits of microscale built environments using online imagery had good reliability with local in-field audits and excellent reliability with local online audits. Results generally supported remote online environmental audits as comparable to local online audits. This identification of low-cost and efficient data acquisition methods is important for expanding research on microscale built environments and physical activity globally.

## Introduction

Greater international attention is being paid to the role the built environment has on physical activity, obesity, and cardiometabolic health [1, 2]. The link between the built environment and physical activity has been well-established using macroscale environmental factors such as street connectivity, land use mix, net-residential density, and composite walkability indices [3–8]. Hundreds of studies to date have been conducted documenting both associations [9] and causal impacts of improvements to the walking environment on utilitarian physical activity [10].

There is a growing awareness that changes to microscale features that enhance the pedestrian walking environment may promote increased physical activity, especially utilitarian physical activity, and are less costly than larger neighborhood and regional scale infrastructure investments [11–13]. Microscale environmental features comprise detailed design characteristics (both quantity and quality) along street block faces or segments (e.g., street amenities like benches and bicycle racks, presence of trees, building setbacks), sidewalks, intersection configuration (e.g., curbs, crosswalks, signalization), types of land use (e.g., residential, commercial, industrial) and traits of the local social environment (e.g., litter, graffiti, and landscaping maintenance) [12]. Observational audits have been a reliable method to gather detailed information on the presence and quality of micro-environment features believed to be relevant for travel behavior, including mode choice, which are not generally available in Geographic Information System (GIS) data furnished by planning agencies [14–16]. In-field data collection requires significant resources to have observers on-site, with staff time encompassing the largest cost. Expenses may also include travel to and from the site, lodging, survey equipment, and transportation between audit locations. In-field data collection may also be adversely impacted by local environmental conditions, such as high crime, traffic-related safety conditions or air pollution, and unfavorable weather conditions, including inclement weather and extreme heat or cold. These expenses and local conditions can limit the scale of research on microscale built environments globally [17].

To overcome many of these limitations, researchers have used free online resources to perform “virtual audits” [17]. Large quantities of public data are available that are suitable to evaluate the built environment through omnidirectional imagery and photogrammetric image interpretation, such as Google Earth and Street View (Google, Inc., Mountain View, CA) and Bing Streetside (Microsoft, Redmond, WA). The imagery made available through these data tools has emerged in the last decade as a viable alternative when auditing general land-use and transportation physical environment characteristics, as well as fine-grain measurements or observing qualitative characteristics, such as sidewalk quality, street furniture, crossing amenities, and curb quality [18–24]. The more recent release of high-definition imagery, coupled with the expansion of coverage to cities around the world, has made online tools and imagery more feasible for use of virtual environmental audits in international studies [16, 25, 26].

The Microscale Audit of Pedestrian Streetscapes (MAPS) is one of several observational tools used to systematically measure microscale built environment features [11, 12, 17, 27]. The MAPS instrument was originally developed in the United States for in-field audits by observers who physically traveled to site locations and performed the audit by walking each route. The MAPS tool has undergone several iterations for different purposes, including creating MAPS-Abbreviated for use in academic research and MAPS-Mini designed to be used by practitioners [17, 28, 29]. The present study implemented the MAPS-Global version, which was developed for international use to represent diverse environments worldwide [11, 16]. While traditionally used as an in-field audit tool, for this study the MAPS-Global audits were completed using both in-field and virtual methods. Zhu et al. and Phillips et al. in the United States, and Vanwolleghem et al. in Belgium have performed studies to test the inter-rater reliability of the MAPS tool using data collected online and found relatively high levels of consistency between observers [16, 17, 30].

Cain et al. also studied the inter-rater reliability of the MAPS-Global tool for local in-field observers in multiple countries [11]. For international studies, local observations require the training of multiple teams of observers who are overseen by different supervisors. These methods have the potential to reduce the comparability of results across countries. An alternative is a centralized data collection that would be remote from most study sites. Possible limitations of this approach include online imagery from different time periods, an inability to read signage (due to clarity of image or language barriers) that identify commercial establishments and a lack of knowledge of some features’ local meaning.

**Fig. 1:**
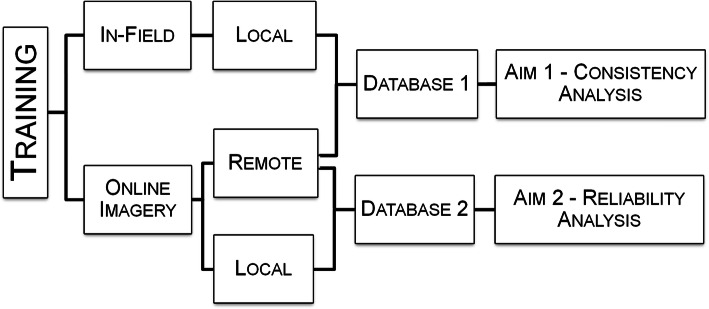
Conceptual model illustrating the development of MAPS-Global databases used to analyze the two study aims

To the knowledge of the authors, there appears to be no study that compares observed microscale environmental conditions recorded by *local* (in-country) observers and *remote* (out of country) observers. The purpose of the present study was to (1) determine consistency in responses of micro-environment observations between local in-field observers and remote online observers, and (2) to measure the reliability of local online observers and remote online observers using the MAPS-Global tool to characterize the pedestrian-related microscale characteristics along streets. The former evaluates the level of correspondence between survey data acquired using two different methods, while the latter examines the same data collected using the same method but by observers with and without local knowledge. Figure 1 provides a conceptual model for the analysis workflow, which applied a systematic training protocol to all observer groups and developed two core comparison databases to analyze the study aims. The study evaluated the validation of public access online resources, specifically Google Earth and Google Street View, as a consistent and reliable method for implementing a virtual MAPS-Global data acquisition without requiring prior knowledge of the local environment.

## Methods

### Residential Addresses

This study used cross-sectional microscale built environment data primarily acquired as part of the International Physical Activity and Environment Network (IPEN) Adolescent study. This international study of adolescents, ages 12 to 16 years, was conducted in 15 countries to ensure a broad range of environments and to maximize variation in participant characteristics. A common research design and methodology, with objective and self-reported measures of physical activity and the built environment, was used to increase intra-regional and inter-country comparability. The principal goal of the study was to evaluate associations of built and social environment features with physical activity, sedentary behavior, and weight status, and then use the evidence to inform evidence-based, international physical activity policies and interventions to mitigate obesity and other chronic diseases in adolescents [31, 32]. Participant recruitment at each study site was stratified by socioeconomic status and location-based walkability, which have been described in detail in other publications [3, 4, 8, 33–38].

For the present study, data were collected from five cities involved in the IPEN Adolescent study: (1) Melbourne, Australia, (2) Ghent, Belgium, (3) Curitiba, Brazil, (4) Hong Kong, China, and (5) Valencia, Spain. Study sites were selected based on each research institution’s access to resources to undertake the microscale built environment inventory, availability of local expertise and support staff to implement the data acquisition, and willingness to participate. MAPS-Global data were collected by local (in-country) observers using both in-field and online resources, as well as remote (outside of the country, all in the U.S.) observers using only online resources. Data collection took place between November 2014 and June 2015. The dataset from these five sites included the home location neighborhood for 40 adolescents participating in the larger IPEN Adolescent study in each of four countries (Belgium, Brazil, China, and Spain) and 40 residential addresses selected at random from within Statistical Area Level 1 (smallest known census unit due to participant privacy restrictions) across walkability and income areas of Melbourne, Australia. The present study had a total sample of 200 targeted address locations.

### MAPS-Global Instrument

Investigators from each site utilized the MAPS-Global observational audit tool to gather the microscale built environment features required for the evaluation. Local and remote observers were provided uniform training manuals, materials, and webinar presentations prepared in English, and all observers practiced and completed sample routes. Each observer also completed a certification requirement for the MAPS-Global tool. More detailed descriptions of the development and design characteristics of the MAPS tool and the certification requirements have been published elsewhere [11, 12, 27].

The MAPS-Global observation tool is designed to capture a range of pedestrian- and bicycle-oriented environment features for defined areas within an international context. The instrument contains a total of 120 items across four sections: (1) the route section tracks land use characteristics and features along the entire route defined by an origin/destination pair; (2) microscale characteristics at the segment level evaluate block faces between intersections; (3) the crossings section collects intersection information; (4) the cul-de-sac section tracks dead-end or cul-de-sac features. The route and cul-de-sac sections of the tool capture built environment characteristics on both sides of the street. In contrast, the segment and crossing sections mainly assess attributes on one side of the street, simulating environmental exposure between home and a nearby destination. The presence of mid-block pedestrian crossings was identified at the segment level of the MAPS-Global instrument. Although data were acquired for cul-de-sacs, due to the low frequency of occurrence in the sample, with some study areas not having any, they were omitted from the analyses. Destinations and land use, streetscapes, aesthetics, and social characteristics expected to be generally consistent throughout the route (e.g., posted speed limits, social environment, and aesthetics) were obtained through the route section. Segment-level measures that evaluate characteristics that were more likely to change throughout the route included sidewalk characteristics, buffers with streets, trees, building configurations, crosswalk presence, traffic signals, and walk signs. An average MAPS-Global route for this study contained 3.2 segment sections and 2.3 crossing sections. When reporting at the route level, the mean value for each variable in these sections was used for routes with multiple segments and crossings.

### Route Selection & Data Acquisition

Residential addresses are a widely investigated location for quantifying built environment exposure within the physical activity literature; thus a residential address point was selected as the most suitable origin for assessment of microscale environments [39, 40]. The microscale environment was observed over a 400 to 725 m (0.25 to 0.45 mile) network distance route along the road network accessible by pedestrians from the residential address toward the nearest identified commercial “cluster” containing at least three businesses within close proximity [12]. The shortest walkable routes along the street network were manually digitized and measured in Google Earth from the origin address points to the nearest commercial cluster for 33 of the 40 MAPS-Global routes per study site.

Online observers used satellite imagery and Google Street View multi-view panoramic road imagery in Google Earth to ensure data were collected for the same route by multiple raters. The vintage of road imagery used for the inventory varied by site depending on availability and update frequency, but was aligned as closely as possible to the time frame of the IPEN Adolescent study. Most of the imagery utilized for the data acquisition by region comprised the following: (1) Melbourne (2014), (2) Ghent (2013), (3) Curitiba (2014), Hong Kong (2011), and Valencia (2014). If the route reached the destination before the minimum distance was reached, additional route segments were added beyond the destination until the minimum threshold (400 m) was surpassed. The average route required 20 min to complete for both in-field and online acquisition methods, however, completion time did vary based on route distance and complexity. Secondarily, single road segment routes located at commercial cluster destinations with crossings at either end (i.e., commercial “blocks”) were also surveyed for a randomly selected sample of seven address locations for each country to provide a wider breadth of environmental features in commercial areas (Table 1). These commercial blocks were determined using satellite imagery and point of interest commercial location information from Google Earth of the closest street network commercial cluster to the address points. A total of 200 routes comprising 649 segments and 459 crossings were collected in the pooled dataset. In each country, 33 residential routes and seven commercial blocks were surveyed, yielding 165 residential routes and 35 commercial blocks for the five-country data set.

**Table 1:** MAPS-Global sample sizes by country and survey component

Country	City	Residential Routes^a^	Commercial Blocks	Survey Component^b^		
				**Total Routes**	**Segments**^**c,d**^	**Crossings**^**d**^
Australia	Melbourne	33	7	40	110	54
Belgium	Ghent	33	7	40	132	94
Brazil	Curitiba	33	7	40	133	107
China	Hong Kong	33	7	40	114	70
Spain	Valencia	33	7	40	160	134
		165 (82.5 %)	35 (17.5 %)			
**Total**				**200**	**649**	**459**

^a^ residential only, commercial blocks not included

^b^ cul-de-sacs were not incorporated into the reliability analysis due to low frequency

^c^ segments are defined as the area between intersections

^d^ both residential and commercial blocks included

Three groups of raters (two local groups for each country and one remote group in the U.S.) recorded microscale built environment data for selected routes using the MAPS-Global instrument. Among the local groups, one used in-field data collection tools, while the other used only online resources. The remote group in the U.S. consisted of two auditors who surveyed all 200 routes online. The local, in-field observers completed the audit using paper surveys, recording observations by pen as they walked the route. Recorded survey forms were then manually entered into a Microsoft Access database in preparation for analysis. For the local and remote groups virtually completing the inventory, Google Earth was used for mapping, route information, aerial imagery, and Google Street View was used for street-level point-of-view imagery. Data were collected independently by in-country and remote observers with no contact or awareness of each other’s results. Each online-rater utilized a dual-monitor workstation to facilitate simultaneous visualization of the microscale environment by virtually walking the route while entering MAPS-Global responses into a database. Data entry raters and project coordinators performed a systematic review of databases for missing values, valid values, and logical consistency among answers. Any rare missing sections or erroneous entries identified were returned to the original observers for review and were resolved before data aggregation and merging into a pooled database was performed. The analyses presented in this study were performed on the pooled database of routes (*n* = 200) in five study regions. By using the pooled database, the analysis allows for a reasonably large sample from which to compute inter-rater reliability and level of consistency; however, it does not offer an opportunity to examine how countries compared with one another

### Instrument Scoring & Subscales

A pooled analysis was performed using MAPS-Global data from all countries together. Variables designed to evaluate similar microscale environmental features were grouped into subscales. This methodology follows the process described in other MAPS and MAPS-Global studies [11, 12, 27]. Briefly, a tiered scoring system was created to summarize item-level variables into subscales at multiple aggregation levels to develop positive and negative valence scores derived from the expected effect of presence, absence, and quality of microscale features on physical activity. For instance, the sum of land uses and destinations supportive of activity-friendly environments, such as mixed-use buildings, access to shops, services, restaurants, and entertainment, were hypothesized to be positively associated with physical activity. In contrast, the presence of physical and social disorder, such as buildings and landscapes not being maintained, littering, and graffiti, was hypothesized to be negatively associated [27]. Cross-domain scores were also computed by summing item-level scores from across all four sections of the instrument to calculate three primary measures of interest: (1) pedestrian infrastructure, (2) pedestrian design, and (3) bicycle facilities. Lastly, overall positive association and negative association scores were produced for segments (positive valence segment scores minus negative valence segments scores) and crossings (positive valence crossing scores minus negative valence crossing scores). Overall meta positive and negative scores were calculated by summing each of the respective positive and negative valence scores from each section (segments and crossings). The overall grand score was derived by subtracting the overall meta negative score from the overall meta positive score.

### Statistical Analyses

The intraclass correlation coefficient (ICC) statistic was used to quantify the level of consistency between MAPS-Global scores derived from the remote (non-local) online assessments with the local in-field assessments and the inter-rater reliability between MAPS-Global scores derived from the local and remote online assessment [41, 42]. Detailed reviews of the analysis methods used for estimating consistency between assessment modes have been published in similar IPEN-focused studies [11, 12, 17, 27, 30]. ICCs were computed using a one-way random model for average measures with a 95 % confidence interval using IBM SPSS Version 21 (IBM Corporation, Armonk, NY) “Reliability Analysis” module [12, 43]. Cicchetti’s ICC numerical ranges and descriptors were used for test-retest reliability for this study: “excellent” (ICC ≥ 0.75), “good” (ICC = 0.60–0.74), “fair” (ICC = 0.40–0.59) and “poor” (ICC < 0.40) [12, 43, 44].

## Results

### In-Country In-Field vs. Remote Online

This section discusses the pooled sample results for the local in-field observers versus remote online observers. The following section reviews the local online observers versus remote online observers. Table 2 shows the ICCs for the local in-field versus remote online comparison. The table includes upper and lower limits for 95 % confidence intervals and descriptive statistics for key MAPS-Global variables comprising route, segment, crossing section measures, section subscales, cross-domain subscales, valence, and grand scores. Descriptive statistics specify the number of individual items included and the range of potential scores for each subscale, central tendency for each subscale, and the frequency and percentage of zero values for each subscale.

**Table 2:** MAPS-Global item-level and subscale levels of consistency and descriptive statistics for in-country in-field observers versus remote online observers

Variable Description^a^	# items (range of scores)	Mean (S.D.)	Null Count (%)	ICC, CI (95 % Lower & Upper Bound)	Sample items and overall subscale description
		**Destinations & Land Use (DLU)**			
**Positive Destinations & Land Use**					
Residential Mix	4 (0–3)	†: 2.60 (1.00) ‡: 2.89 (0.85)	5 (2.6 %) 3 (1.5 %)	0.47 (0.35, 0.57)	Single family, multi-family, mixed, apartment over retail
Shops	8 (0–28)	†: 5.12 (5.65) ‡: 4.02 (4.11)	57 (29.1 %) 57 (29.1 %)	0.71 (0.68, 0.77)	Grocery, convenience store, bakery, drugstore, other retail, shopping mall, strip mall, open-air market
Restaurant-Entertainment	4 (0–20)	†: 2.74 (3.53) ‡: 2.10 (2.84)	81 (41.3 %) 95 (48.5 %)	0.64 (0.54, 0.71)	Fast food, sit-down, café, entertainment
Institutional-Service	3 (0–15)	†: 4.61 (4.15) ‡: 2.92 (3.34)	43 (21.9 %) 77 (39.3 %)	0.65 (0.56, 0.72)	Bank, health-related professional, other service
Worship	1 (0–5)	†: 0.28 (0.60) ‡: 0.22 (0.61)	148 (75.5 %) 165 (84.2 %)	0.56 (0.46, 0.65)	Place of worship
School	1 (0–5)	†: 0.87 (1.27) ‡: 0.34 (0.58)	95 (48.5 %) 140 (71.4 %)	0.18 (0.04, 0.37)	School land use
Public Recreation	4 (0–20)	†: 0.51 (0.73) ‡: 0.46 (0.75)	120 (61.2 %) 130 (66.3 %)	0.47 (0.35, 0.57)	Public indoor, public outdoor facility, park, trail
Private Recreation	2 (0–10)	†: 0.18 (0.49) ‡: 0.15 (0.40)	169 (86.2 %) 170 (86.7 %)	0.27 (0.14, 0.40)	Private indoor, private outdoor facility
Pedestrian Street	1 (0–5)	†: 0.18 (0.53) ‡: 0.16 (0.46)	155 (79.1 %) 171 (87.2 %)	0.34 (0.21, 0.46)	Pedestrian street/zone
**Negative Destinations & Land Use**					
Age-restricted bar or nightclub	1 (0–5)	†: 0.14 (0.50) ‡: 0.33 (0.78)	140 (74.1 %) 156 (79.6 %)	0.04 (-0.10, 0.18)	Age-restricted bar/nightclub
Liquor or alcohol store	1 (0–5)	†: 0.04 (0.19) ‡: 0.06 (0.26)	180 (91.8 %) 186 (94.9 %)	0.16 (0.02, 0.29)	Liquor or alcohol store
Positive DLU	28 (0-111)	†: 16.46 (13.58) ‡: 12.20 (9.68)	0 (0.0 %) 0 (0.0 %)	0.69 (0.60, 0.75)	Sum of the positive DLU subscales
Negative DLU	2 (0–10)	†: 0.29 (0.82) ‡: 0.44 (0.93)	161 (82.1 %) 143 (73.0 %)	0.06 (-0.08, 0.20)	Sum of the negative DLU subscales
Overall DLU	30	†: 16.18 (13.41) ‡: 11.76 (9.32)	0 (0.0 %) 0 (0.0 %)	0.66 (0.59, 0.75)	Positive DLU - Negative DLU
**Streetscape Characteristics**					
Positive Streetscape	22 (0–29)	†: 7.19 (4.56) ‡: 5.51 (3.88)	15 (7.7 %) 25 (12.8 %)	0.66 (0.58, 0.73)	Transit, traffic calming, trash bins, benches, bike racks, bike lockers, bike docking stations, kiosks, hawkers.
**Aesthetics & Social Characteristics**					
Positive Aesthetics/Social	4 (0–4)	†: 0.81 (0.84) ‡: 1.56 (1.15)	82 (41.8 %) 47 (24.0 %)	0.09 (-0.05, 0.23)	Hardscape, water, softscape, landscaping
Negative Aesthetics/Social	6 (0–6)	†: 2.86 (1.44) ‡: 1.76 (1.50)	12 (6.1 %) 57 (29.1 %)	0.16 (0.02, 0.30)	Buildings not maintained, graffiti, litter, dog fouling, physical disorder, highway near
Overall Aesthetics/Social	10	†: -2.05 (1.81) ‡: -0.19 (2.42)	16 (8.2 %) 24 (12.2 %)	0.11 (-0.03, 0.24)	Positive Aesthetics/Social - Negative Aesthetics/Social
**Crossings/Intersections**					
**Positive Crossing Subscales**					
Crosswalk Amenities	7 (0–7)	†: 0.88 (0.93) ‡: 1.03 (1.09)	69 (38.8 %) 76 (42.7 %)	0.85 (0.80, 0.88)	Crossing aids, marked crosswalk, high visibility striping, different material, curb extension, raised crosswalk, refuge islands
Curb Quality & Presence	3 (0–6)	†: 4.24 (1.42) ‡: 3.33 (2.09)	3 (1.7 %) 24 (13.5 %)	0.53 (0.41, 0.62)	Curb presence, curb ramps lined up, tactile paving
Intersection Control & Signage	7 (0–7)	†: 1.03 (0.82) ‡: 1.08 (0.79)	26 (14.6 %) 27 (15.2 %)	0.82 (0.77, 0.87)	Yield signs, stop signs, traffic signal, traffic circle, pedestrian walk signals, push buttons, countdown signal
Bicycle Features	3 (0–3)	†: 0.04 (0.17) ‡: 0.04 (0.12)	168 (94.4 %) 163 (91.6 %)	0.65 (0.55, 0.72)	Waiting area, bike lane crossing the crossing, bike signal
Overpass	1 (0–1)	†: 0.01 (0.08) ‡: 0.04 (0.16)	185 (97.4 %) 178 (93.7 %)	0.02 (-0.12, 0.17)	Crossing on pedestrian overpass, bridge
**Negative Crossing Subscales**					
Road Width	1 (0–2)	†: 0.19 (0.42) ‡: 0.54 (0.54)	73 (41.0 %) 141 (79.2 %)	0.40 (0.26, 0.51)	Distance of crossing leg
Positive Crossing	21 (0–24)	†: 6.20 (2.78) ‡: 5.49 (3.35)	2 (1.1 %) 7 (3.9 %)	0.82 (0.77, 0.87)	Sum of the positive crossing subscales
Negative Crossing	1 (0–2)	†: 0.19 (0.42) ‡: 0.54 (0.54)	73 (41.0 %) 141 (79.2 %)	0.40 (0.26, 0.51)	Sum of the negative crossing subscales
Overall Crossing	22	†: 6.01 (2.54) ‡: 4.94 (3.27)	2 (1.1 %) 1 (0.6 %)	0.76 (0.69, 0.82)	Positive Crossing - Negative Crossing
**Street Segments**					
**Positive Segment Subscales**					
Building Height-Setback	4 (0–10)	†: 6.11 (3.71) ‡: 5.83 (3.13)	0 (0.0 %) 0 (0.0 %)	0.84 (0.80, 0.88)	Building height, smallest and largest setback
Building Height-Road Width Ratio	5 (0–3)	†: 1.17 (1.06) ‡: 1.20 (0.91)	55 (30.2 %) 25 (13.7 %)	0.56 (0.45, 0.65)	Building height, setback and road width
Buffer	2 (0–5)	†: 3.01 (1.61) ‡: 2.80 (1.38)	18 (10.0 %) 11 (6.1 %)	0.40 (0.27, 0.51)	Parking along street, buffer
Bike Infrastructure	3 (0–5)	†: 0.29 (1.08) ‡: 0.33 (0.88)	178 (95.2 %) 153 (81.8 %)	0.57 (0.47, 0.66)	Bike lane presence, quality, signage
Shade	3 (0–6)	†: 0.16 (0.41) ‡: 0.11 (0.32)	163 (85.8 %) 168 (88.4 %)	0.76 (0.69, 0.81)	Number of trees, sidewalk coverage, shade
Sidewalk	2 (0–6)	†: 19.55 (6.18) ‡: 18.81 (4.70)	0 (0.0 %) 0 (0.0 %)	0.76 (0.69, 0.81)	Sidewalk presence and width
Pedestrian infrastructure	5 (0–5)	†: 1.07 (0.78) ‡: 1.22 (0.55)	0 (0.0 %) 0 (0.0 %)	0.39 (0.26, 0.51)	Mid-segment crossing, pedestrian bridge, covered place to walk, street lights
Building Aesthetics and Design	1 (0–2)	†: 1.34 (0.67) ‡: 1.19 (0.67)	25 (13.2 %) 22 (11.6 %)	0.53 (0.42, 0.63)	Street windows
Informal Path or Shortcut	1 (0–1)	†: 0.13 (0.26) ‡: 0.02 (0.10)	160 (84.2 %) 187 (98.4 %)	0.07 (-0.07, 0.21)	Informal path connecting to something else
Hawkers/Shops	1 (0–2)	†: 0.14 (0.43) ‡: 0.00 (0.02)	172 (90.5 %) 190 (100.0 %)	-0.03 (0.17, 0.11)	Hawkers/shops on sidewalk/pedestrian zone
**Negative Segment Subscales**					
Sidewalk	7 (0–13)	†: 3.31 (3.39) ‡: 2.24 (1.75)	33 (17.7 %) 20 (10.8 %)	0.28 (0.15, 0.41)	Non-continuous sidewalk, trip hazards, obstructions, cars blocking walkway, slope, gates, driveways
Positive Segment	27 (0–45)	†: 19.55 (6.18) ‡: 18.81 (4.70)	0 (0.0 %) 0 (0.0 %)	0.76 (0.69, 0.81)	Sum of the positive segment subscales
Negative Segment	7 (0–13)	†: 3.21 (2.49) ‡: 2.25 (1.75)	11 (5.9 %) 27 (14.4 %)	0.52 (0.41, 0.62)	Sum of the negative segment subscales
Overall Segment	34	†: 16.15 (8.56) ‡: 16.54 (5.95)	0 (0.0 %) 0 (0.0 %)	0.73 (0.65, 0.79)	Positive Segment - Negative Segment
**Overall Valence and Grand Scores**					
Overall Positive	102 (0-210)	†: 10.04 (5.59) ‡: 8.71 (4.55)	0 (0.0 %) 0 (0.0 %)	0.60 (0.52, 0.68)	Positive DLU, positive streetscape, positive aesthetics/social, positive segment (mean of all segments), positive crossing (mean of all segments).
Overall Negative	16 (0–22)	†: 1.63 (1.29) ‡: 1.25 (1.18)	11 (5.6 %) 27 (13.8 %)	0.29 (0.15, 0.41)	Negative DLU, negative aesthetics/social, negative segment (mean of all segments), negative crossing (mean of all crossings).
Overall Grand Score	118	†: 22.08 (15.55) ‡: 18.21 (11.17)	0 (0.0 %) 0 (0.0 %)	0.75 (0.68, 0.80)	Overall Positive - Overall Negative
**Cross-Domain Subscales**					
Pedestrian Infrastructure	13 (0–27)	†: 7.90 (2.51) ‡: 7.48 (2.41)	3 (1.7 %) 2 (1.1 %)	0.69 (0.60–0.76)	Trail, pedestrian zone, sidewalk presence/width, buffer, shortcut, mid-segment crossing, pedestrian bridge, air-conditioned place to walk, low lights, overpass, crosswalk, refuge island
Pedestrian Design	13 (0–21)	†: 10.23 (4.00) ‡: 8.76 (4.00)	1 (0.6 %) 0 (0.0 %)	0.82 (0.76–0.86)	Open-air market, trash cans, benches, kiosks, hawkers and shops, setback, visibility, pedestrian walk signals, push buttons, countdown signals, ramps, crossing aids
Bicycle Facilities	9 (0–11)	†: 0.78 (1.31) ‡: 0.77 (1.19)	119 (68.0 %) 97 (55.4 %)	0.73 (0.65–0.79)	Bike racks, docking stations, lockers, bike lane, bike lane quality, signs, bike signal, bike box, bike lane perpendicular to the crossing

^a^: Cul-de-sac/Dead-end variables were excluded due to low frequency

†: In-country, in-field observer

‡: Remote, online observer

The overall grand score had an ICC of 0.75 (95 % confidence interval: 0.68–0.80). Positive subscales performed much better than negative subscales. The exceptions were positive aesthetics/social characteristics (ICC = 0.09), overpass (ICC = 0.02), informal path or shortcut (ICC = 0.07), and hawkers/shops (ICC = -0.03), which performed poorly. Positive subscales for destinations and land-use components (ICC = 0.69) from the route section, as well as positive crossing subscales (ICC = 0.82) and positive street segment subscales (ICC = 0.76), also performed well, producing an acceptable ICC of 0.60 for the overall positive valence score. Among individual-item MAPS-Global components, building height-setback from the street and intersection controls performed the strongest with ICC values of 0.84 and 0.82, respectively. Sidewalks (ICC = 0.76) and streetscape characteristics (ICC = 0.66) also provided ICC values in the “good” to “excellent” range. The positive cross-domain subscales all performed well, with ICC values in the “good” to “excellent” range. Pedestrian design yielded a high ICC at 0.82. Bicycle facilities also yielded a relatively high ICC of 0.73, despite a majority of observed routes not including these features.

### In-Country Online vs. Remote Online

Table 3 shows ICC results and descriptive statistics for the local online versus remote online comparisons. The overall grand score had an ICC of 0.91 (95 % confidence interval: 0.88–0.93). Similar to the local in-field versus remote online analysis, positive subscales yielded stronger results in comparison to negative subscales, except for the similarly poor-performing positive aesthetics/social characteristics (ICC = 0.21), overpass (ICC = 0.24), and hawkers (street vendors)/shops (ICC = 0.002) variables. Positive subscales for destinations and land-use components (ICC = 0.92) from the route section, as well as positive crossing subscales (ICC = 0.75) and positive street segment subscales (ICC = 0.71), also performed well, producing an acceptable overall positive valence score of 0.64. Among individual-level MAPS-Global components, count of shops (ICC = 0.89), restaurants (ICC = 0.85), and institutional-services (ICC = 0.84) from the destination and land use section, and crosswalk amenities (ICC = 0.81) from the crossing section performed the strongest. With an ICC of 0.75, building height-setback also provided ICC values in the “excellent” range. Positive subscales such as intersection control and signage (ICC = 0.72), building height-road width ratio (ICC = 0.63), sidewalk (ICC = 0.62), and public recreation (ICC = 0.60) also performed well, yielding ICCs in the “good” range. The positive cross-domain subscales performed well in the “good” to “excellent” range with bicycle facilities showing a relatively high ICC of 0.80, and pedestrian design and pedestrian infrastructure performing well with ICCs of 0.74 and 0.65, respectively.

**Table 3:** MAPS-Global item-level and subscale inter-rater reliability and descriptive statistics for in-country online observers versus remote online observers

Variable Description^a^	# items (range of scores)	Mean (S.D.)	Null Count (%)	ICC, CI (95 % Lower & Upper Bound)	Sample items and overall subscale description
		**Destinations & Land Use (DLU)**			
**Positive Destinations & Land Use**					
Residential Mix	4 (0–3)	†: 2.62 (0.99) ‡: 2.89 (0.85)	3 (1.5 %) 3 (1.5 %)	0.48 (0.37, 0.58)	Single family, multi-family, mixed, apartment over retail
Shops	8 (0–28)	†: 4.10 (4.70) ‡: 4.02 (4.11)	60 (30.5 %) 57 (29.1 %)	0.89 (0.86, 0.92)	Grocery, convenience store, bakery, drugstore, other retail, shopping mall, strip mall, open-air market
Restaurant-Entertainment	4 (0–20)	†: 4.10 (4.70) ‡: 2.10 (2.84)	93 (47.2 %) 95 (48.5 %)	0.85 (0.81, 0.89)	Fast food, sit-down, café, entertainment
Institutional-Service	3 (0–15)	†: 3.67 (3.83) ‡: 2.92 (3.34)	61 (31.0 %) 77 (39.3 %)	0.84 (0.79, 0.88)	Bank, health-related professional, other service
Worship	1 (0–5)	†: 0.19 (0.48) ‡: 0.22 (0.61)	166 (84.3 %) 165 (84.2 %)	0.56 (0.46, 0.65)	Place of worship
School	1 (0–5)	†: 0.55 (0.84) ‡: 0.34 (0.58)	120 (60.9 %) 140 (71.4 %)	0.50 (0.38, 0.59)	School land use
Public Recreation	4 (0–20)	†: 0.49 (0.81) ‡: 0.46 (0.75)	130 (66.3 %) 130 (66.3 %)	0.60 (0.50, 0.68)	Public indoor, public outdoor facility, park, trail
Private Recreation	2 (0–10)	†: 0.16 (0.48) ‡: 0.15 (0.40)	173 (87.8 %) 170 (86.7 %)	0.27 (0.13, 0.39)	Private indoor, private outdoor facility
Pedestrian Street	1 (0–5)	†: 0.22 (0.65) ‡: 0.16 (0.46)	170 (86.7 %) 171 (87.2 %)	0.45 (0.33, 0.56)	Pedestrian street/zone
**Negative Destinations & Land Use**					
Age-restricted bar or nightclub	1 (0–5)	†: 0.13 (0.42) ‡: 0.33 (0.78)	177 (89.8 %) 156 (79.6 %)	0.17 (0.03, 0.31)	Age-restricted bar/nightclub
Liquor or alcohol store	1 (0–5)	†: 0.04 (0.26) ‡: 0.06 (0.26)	190 (96.4 %) 186 (94.9 %)	0.39 (0.27, 0.50)	Liquor or alcohol store
Positive DLU	28 (0-111)	†: 13.50 (11.49) ‡: 12.20 (9.68)	0 (0.0 %) 0 (0.0 %)	0.92 (0.89, 0.93)	Sum of the positive DLU subscales
Negative DLU	2 (0–10)	†: 0.35 (0.95) ‡: 0.44 (0.93)	158 (80.2 %) 143 (73.0 %)	0.30 (0.17, 0.42)	Sum of the negative DLU subscales
Overall DLU	30	†: 13.16 (11.50) ‡: 11.76 (9.32)	0 (0.0 %) 0 (0.0 %)	0.89 (0.86, 0.92)	Positive DLU - Negative DLU
**Streetscape Characteristics**					
Positive Streetscape	22 (0–29)	†: 6.26 (3.81) ‡: 5.51 (3.88)	12 (6.1 %) 25 (12.8 %)	0.62 (0.52, 0.69)	Transit, traffic calming, trash bins, benches, bike racks, bike lockers, bike docking stations, kiosks, hawkers.
**Aesthetics & Social Characteristics**					
Positive Aesthetics/Social	4 (0–4)	†: 0.99 (0.83) ‡: 1.56 (1.15)	57 (28.9 %) 47 (24.0 %)	0.21 (0.08, 0.34)	Hardscape, water, softscape, landscaping
Negative Aesthetics/Social	6 (0–6)	†: 2.13 (1.46) ‡: 1.76 (1.50)	37 (18.8 %) 57 (29.1 %)	0.54 (0.43, 0.63)	Buildings not maintained, graffiti, litter, dog fouling, physical disorder, highway near
Overall Aesthetics/Social	10	†: -1.13 (1.91) ‡: -0.19 (2.42)	30 (15.2 %) 24 (12.2 %)	0.47 (0.35, 0.57)	Positive Aesthetics/Social - Negative Aesthetics/Social
**Crossings/Intersections**					
**Positive Crossing Subscales**					
Crosswalk Amenities	7 (0–7)	†: 1.17 (1.38) ‡: 1.03 (1.09)	92 (51.9 %) 76 (42.7 %)	0.81 (0.78, 0.84)	Crossing aids, marked crosswalk, high visibility striping, different material, curb extension, raised crosswalk, refuge islands
Curb Quality & Presence	3 (0–6)	†: 4.11 (1.78) ‡: 3.33 (2.09)	16 (9.0 %) 24 (13.5 %)	0.46 (0.38, 0.53)	Curb presence, curb ramps lined up, tactile paving
Intersection Control & Signage	7 (0–7)	†: 0.95 (1.06) ‡: 1.08 (0.79)	74 (41.6 %) 27 (15.2 %)	0.73 (0.68, 0.77)	Yield signs, stop signs, traffic signal, traffic circle, pedestrian walk signals, push buttons, countdown signal
Bicycle Features	3 (0–3)	†: 0.04 (0.24) ‡: 0.04 (0.12)	172 (96.5 %) 163 (91.6 %)	0.48 (0.41, 0.55)	Waiting area, bike lane crossing the crossing, bike signal
Overpass	1 (0–1)	†: 0.01 (0.09) ‡: 0.04 (0.16)	171 (96.1 %) 178 (93.7 %)	0.24 (0.16 0.33)	Crossing on pedestrian overpass, bridge
**Negative Crossing Subscales**					
Road Width	1 (0–2)	†: 0.14 (0.44) ‡: 0.54 (0.54)	159 (89.5 %) 141 (79.2 %)	0.39 (0.31, 0.46)	Distance of crossing leg
Positive Crossing	21 (0–24)	†: 6.34 (3.60) ‡: 5.49 (3.35)	11 (6.3 %) 7 (3.9 %)	0.75 (0.71, 0.79)	Sum of the positive crossing subscales
Negative Crossing	1 (0–2)	†: 0.14 (0.44) ‡: 0.54 (0.54)	159 (89.5 %) 141 (79.2 %)	0.39 (0.31, 0.46)	Sum of the negative crossing subscales
Overall Crossing	22	†: 6.22 (3.45) ‡: 4.94 (3.27)	11 (6.2 %) 1 (0.6 %)	0.70 (0.65, 0.75)	Positive Crossing - Negative Crossing
**Street Segments**					
**Positive Segment Subscales**					
Building Height-Setback	4 (0–10)	†: 6.00 (3.33) ‡: 5.83 (3.13)	0 (0.0 %) 0 (0.0 %)	0.75 (0.72, 0.78)	Building height, smallest and largest setback
Building Height-Road Width Ratio	5 (0–3)	†: 1.24 (1.07) ‡: 1.20 (0.91)	51 (28.0 %) 25 (13.7 %)	0.63 (0.58, 0.67)	Building height, setback and road width
Buffer	2 (0–5)	†: 3.24 (1.78) ‡: 2.80 (1.38)	23 (12.6 %) 11 (6.1 %)	0.58 (0.53, 0.63)	Parking along street, buffer
Bike Infrastructure	3 (0–5)	†: 0.36 (1.16) ‡: 0.33 (0.88)	41 (21.4 %) 153 (81.8 %)	0.58 (0.76, 0.81)	Bike lane presence, quality, signage
Shade	3 (0–6)	†: 1.70 (1.39) ‡: 0.11 (0.32)	41 (21.4 %) 168 (88.4 %)	0.53 (0.47, 0.58)	Number of trees, sidewalk coverage, shade
Sidewalk	2 (0–6)	†: 5.14 (1.19) ‡: 18.81 (4.70)	5 0 (5.5 %) 0 (0.0 %)	0.62 (0.57, 0.66)	Sidewalk presence and width
Pedestrian infrastructure	5 (0–5)	†: 0.91 (0.71) ‡: 1.22 (0.55)	0 (0.0 %) 0 (0.0 %)	0.19 (0.11, 0.26)	Mid-segment crossing, pedestrian bridge, covered place to walk, street lights
Building Aesthetics and Design	1 (0–2)	†: 0.73 (0.82) ‡: 1.19 (0.67)	96 (50.3 %) 22 (11.6 %)	0.43 (0.36, 0.49)	Street windows
Informal Path or Shortcut	1 (0–1)	†: 2.19 (0.90) ‡: 0.02 (0.10)	165 (86.9 %) 187 (98.4 %)	0.49 (0.42, 0.54)	Informal path connecting to something else
Hawkers/Shops	1 (0–2)	†: 0.18 (0.67) ‡: 0.00 (0.02)	183 (96.1 %) 190 (100.0 %)	0.002 (-0.08, 0.08)	Hawkers/shops on sidewalk/pedestrian zone
**Negative Segment Subscales**					
Sidewalk	7 (0–13)	†: 2.64 (2.05) ‡: 2.24 (1.75)	35 (19.0 %) 20 (10.8 %)	0.65 (0.62, 0.68)	Non-continuous sidewalk, trip hazards, obstructions, cars blocking walkway, slope, gates, driveways
Positive Segment	27 (0–45)	†: 20.16 (6.05) ‡: 18.81 (4.70)	0 (0.0 %) 0 (0.0 %)	0.73 (0.69, 0.76)	Sum of the positive segment subscales
Negative Segment	7 (0–13)	†: 2.64 (2.05) ‡: 2.25 (1.75)	36 (19.7 %) 27 (14.4 %)	0.71 (0.68, 0.74)	Sum of the negative segment subscales
Overall Segment	34	†: 17.50 (7.02) ‡: 16.54 (5.95)	0 (0.0 %) 0 (0.0 %)	0.77 (0.74, 0.80)	Positive Segment - Negative Segment
**Overall Valence and Grand Scores**					
Overall Positive	102 (0-210)	†: 9.45 (5.16) ‡: 8.71 (4.55)	0 (0.0 %) 0 (0.0 %)	0.64 (0.57, 0.70)	Positive DLU, positive streetscape, positive aesthetics/social, positive segment (mean of all segments), positive crossing (mean of all segments).
Overall Negative	16 (0–22)	†: 1.31 (1.23) ‡: 1.25 (1.18)	37 (18.8 %) 27 (13.8 %)	0.48 (0.39, 0.57)	Negative DLU, negative aesthetics/social, negative segment (mean of all segments), negative crossing (mean of all crossings).
Overall Grand Score	118	†: 19.65 (12.47) ‡: 18.21 (11.17)	0 (0.0 %) 0 (0.0 %)	0.91 (0.88, 0.93)	Overall Positive - Overall Negative
**Cross-Domain Subscales**					
Pedestrian Infrastructure	13 (0–27)	†: 8.52 (2.43) ‡: 7.48 (2.41)	0 (0.0 %) 2 (1.1 %)	0.65 (0.55–0.72)	Trail, pedestrian zone, sidewalk presence/width, buffer, shortcut, mid-segment crossing, pedestrian bridge, air-conditioned place to walk, low lights, overpass, crosswalk, refuge island
Pedestrian Design	13 (0–21)	†: 10.06 (4.01) ‡: 8.76 (4.00)	0 (0.0 %) 0 (0.0 %)	0.74 (0.67–0.80)	Open-air market, trash cans, benches, kiosks, hawkers and shops, setback, visibility, pedestrian walk signals, push buttons, countdown signals, ramps, crossing aids
Bicycle Facilities	9 (0–11)	†: 0.83 (1.32) ‡: 0.77 (1.19)	102 (58.0 %) 97 (55.4 %)	0.80 (0.74–0.85)	Bike racks, docking stations, lockers, bike lane, bike lane quality, signs, bike signal, bike box, bike lane perpendicular to the crossing

^a^: Cul-de-sac/Dead-end variables were excluded due to low frequency

†: In-country, online observer

‡: Remote, online observer

## Discussion

This study measured (1) the consistency in responses between local in-field observers and non-local remote online observers and (2) the reliability between in-country online observers and non-local remote online observers using the MAPS-Global tool for walking routes from residential addresses to the nearest commercial cluster in five countries: Australia, Belgium, Brazil, China, and Spain. The ICCs of the two comparison analyses showed relatively high consistency among observers. Moderately stronger results were observed for the local online and remote online sample (ICC grand score = 0.91) versus the local in-field and remote online data collection analysis (ICC grand score = 0.75). This pattern indicates a higher consistency among raters using a similar methodology and images to review when applying the MAPS-Global tool. The online method did not result in any substantial loss in accurately completing any of the items in the tool, compared to the in-field method.

Despite the systematic application of the MAPS-Global instrument for both in-field and online observers, inherent differences in data acquisition methods present a potential for variability in the resulting audit scores. Possible discrepancies in observations were also anticipated between local and non-local observers. Local observers were more familiar with the local environment, read the local language (useful when using signage to discern features), and were more accustomed to local facilities, services, and businesses than non-locals. As a result of these expected causes of variability among the data, steps were taken to ensure consistent training and certification for observers. Survey teams from each country participated in training presentations, completed practice audits in groups and independently, and achieved consistency in responses to become certified. Although some observers may have had previous experience with data collection using MAPS-Global or another version of MAPS, both local and remote observers all received a consistent level of training to complete the certification requirements. Local observers may or may not have been directly familiar with the routes they completed. In contrast, remote observers did not know the route areas, nor necessarily the written language used in each region. Therefore, a noteworthy finding of the study was that remote observers could accurately complete the survey assessment using online imagery while not being familiar with microscale environment features in other languages, such as street signs, names of businesses, or civic services, storefront advertising, and transit stops.

Positive subscales for destinations and land-use components from the route section and positive crossing/intersection subscales and positive subscales for street segment subscales showed the strongest levels of alignment between each observer for both analyses. Similar to the results found by Cain et al., both positive and negative aesthetics and social characteristics had low ICC scores for both comparisons, which may reflect the fact that these characteristics often unintentionally introduce more subjectivity in observer responses [11]. Aesthetics and social characteristics have continued to remain part of the MAPS-Global instrument and other MAPS versions because of the sustained interest in having these types of measures available for analysis. Nevertheless, caution is warranted when interpreting the results of these variables. Interestingly, there were some notable differences between the two analyses regarding subscale ICC values. When comparing scoring responses for individual positive subscales, the crossing/intersection sections had the highest consistency levels between local in-field observes and remote online observers. In contrast, the destination and land use section was most reliable for scores between local online and remote online. These differences suggest that, in addition to the level of familiarity with the local environment, the method of data collection (i.e., in-field vs. online) may influence the reliability of subscales.

While cross-domain subscales and positive subscales for destination and land use, streetscape characteristics, crossing/intersections, and street segments were reliable, negative subscales for these sections were not. The negative subscales tended to comprise fewer items, limiting variability in general and the strength of the ICC. In fact, with only a few exceptions, in each section of the tool, microscale features with limited occurrence tended to produce lower ICC values than more commonly observed features. For example, among age-restricted bars, liquor stores, and private recreation, all had relatively low frequencies (null range: 96.4-74.1 %) and performed poorly (ICC range: 0.39–0.04). Weak ICC values were also observed for the presence of overpasses in the crossing section and pedestrian infrastructure, informal paths, and hawkers in the segment section, the latter two items rarely being present. Despite the relatively poorer performance of these features, these less frequently occurring microscale features can have important impacts on the pedestrian environment, though perhaps only in areas where they are more common, which could be low-income countries. These items should continue to be captured as part of the tool, even if that requires increased caution when interpreting their presence.

When using MAPS-Global in international studies, online observations are recommended so long as online imagery quality, currency, and extent are sufficiently high to perform the inventory accurately. Despite some variation in image currency between study sites (2011–2014), all imagery was available in high-definition and provided no barriers to observers’ accurate interpretation. There were some rare instances of image obstruction of features by large vehicles, construction sites, or areas where imagery from all travel lanes was not available. These issues were overcome through further navigation of the route to nearby or adjacent streets or observing features from different angles within the Street View interface. Other tools within the Google Earth software, such as satellite imagery, photos, photosphere images, and spatial measurement tools to assess distances were also used. It is acknowledged that it may be possible that a few of the survey questions about physically small details (e.g., trip hazards or sidewalk heaves) might be missed in the imagery and may be more easily apparent to observers when physically walking the segment.

The current study involved different observers for local in-field and local online data collection, limiting the study’s analyses and the authors’ ability to determine whether differences were more influenced by the individual completing the survey or the method of completing the survey. The inability to apply a random assignment of observers to in-field and online domains due to data acquisition resources was also recognized as a limitation. The study provided international breadth by including data from five countries worldwide, however, the relatively small sample was limited to urbanized areas in five cities. Although consistent training manuals and materials were distributed, and the same certification process was used for each country, the reliability results presented here suggest that future studies should continue to enhance the training protocols to limit variability even further. Researchers may consider an increased unification of training sessions and practice routes among all observers at the same time to ensure that presentation materials and example scenarios are delivered by the same person(s). This will allow all observers an opportunity to become familiar with a broad range of microscale features and the prescribed consistent way to score them, reducing ambiguity and subjectivity of responses. Differences in supervision methods across countries are likely a source of error that may be more difficult to standardize. However, it might be possible for the supervision of observers in all countries to be overseen centrally by the same person, though that protocol was not used in the current study.

## Conclusions

To expand the research and data available on microscale built environments and their implications for physical activity at an international level, there must be a continued concerted effort to identify more cost-effective and efficient methods of data acquisition. Recent research found the MAPS-Global instrument to be a valid and reliable audit tool for in-field data collection of microscale features of the built environment [11]. MAPS-Global can be implemented more broadly using online resources that are rapidly becoming available globally. This study demonstrated a relatively high level of reliability for composite subscale measures, especially pedestrian design and bicycle facilities, and a high level of consistency for grand overall scores of remote online observations compared to both local in-field and local online data collection. Researchers should exercise caution using MAPS-Global for virtual audits, whether by local or remote observers, when interpreting positive and negative subscale scores for aesthetic/social characteristics, and microscale features that are rarely observed. The results presented in this study support the use of remote online observations with MAPS-Global as an effective alternative to local data collection. Using a central team of observers and supervisors to conduct online observations in multiple countries could be an efficient approach to building an international database that maximizes comparability across countries.

## Data Availability

The data presented in this article are not shared publicly because the data are proprietary and owned individually by each participating research institution. Data acquired pertaining to participant health and behavior must be securely protected to ensure participant privacy and cannot be made available to the public.

## Abbreviations

DLU: Destinations and land use
GIS: Geographic information systems
ICC: Intraclass correlation coefficient
IPEN: International Physical Activity and the Environment Network
MAPS: Microscale Audit of Pedestrian Streetscapes
